# Comparative Accuracy of Clinical and Inflammatory Scores for Predicting Mortality and Rebleeding After Acute Gastroesophageal Variceal Bleeding

**DOI:** 10.3390/jcm15134976

**Published:** 2026-06-26

**Authors:** Hakan Demiröz, Yusuf Bünyamin Ketenci, Mehmet Akca, Iyas F. M. Ayyash, Müge Ustaoğlu Dede, Ufuk Avcıoğlu, Talat Ayyıldız, İbrahim Gören, Ahmet Bektaş

**Affiliations:** 1 Department of Gastroenterology, Faculty of Medicine, Ondokuz Mayıs University, Samsun 55270, Turkey; bketenci@gmail.com (Y.B.K.); md.makca@gmail.com (M.A.); ustaoglu.md@gmail.com (M.U.D.); ufukavcioglu@yahoo.com (U.A.); talatayy@gmail.com (T.A.); drigoren@hotmail.com (İ.G.); abektas@omu.edu.tr (A.B.); 2 Department of Internal Medicine, Faculty of Medicine, Ondokuz Mayıs University, Samsun 55270, Turkey; eeyyaass.222@gmail.com

**Keywords:** acute gastroesophageal variceal bleeding, cirrhosis, prognostic scoring, short-term mortality

## Abstract

**Background/Objectives:** Acute gastroesophageal variceal bleeding (AGEVB) is a major cause of mortality and treatment failure in cirrhotic patients. Early risk stratification remains essential for optimizing management and intensive monitoring. To compare the prognostic performance of Child–Pugh (CTP), Model for End-Stage Liver Disease–Na (MELD-Na), platelet count-to-spleen diameter ratio (PC/SD), and neutrophil-to-lymphocyte ratio (NLR) in predicting 6-week mortality and early rebleeding after AGEVB. **Methods:** This retrospective single-center cohort study included 150 cirrhotic patients admitted with acute GEVB. Baseline laboratory and clinical parameters were used to calculate CTP, MELD-Na, PC/SD, and NLR. Predictive accuracy for mortality and rebleeding was evaluated using receiver operating characteristic analysis and penalized multivariable logistic regression. **Results:** Six-week mortality occurred in 11.3% and early rebleeding in 20.0% of patients. CTP and MELD-Na scores were significantly higher among non-survivors and patients with rebleeding. The CTP score demonstrated the highest discriminative performance for both outcomes. NLR and PC/SD showed limited predictive value and were not independent predictors after adjustment. **Conclusions:** Scores reflecting hepatic functional reserve, particularly the Child–Pugh score, provide superior prognostic accuracy compared with inflammatory and portal hypertension markers in AGEVB.

## 1. Introduction

Liver cirrhosis is a global health concern characterized by an increasing burden of morbidity and mortality worldwide. One of the most critical turning points in the natural course of the disease is AGEVB, which serves as a hallmark of clinical decompensation [1]. Despite advancements in endoscopic treatments, vasoactive medications, and prophylactic antibiotic use, 6-week mortality rates among patients with AGEVB remain at approximately 15–20% [2]. Furthermore, the risk of early rebleeding following the initial episode persists as a significant complication that directly threatens survival [3]. Consequently, the early identification of high-risk groups among patients presenting with AGEVB is of vital importance for intensifying treatment strategies and managing intensive care follow-up [4].

In clinical practice, CTP and MELD scores are widely utilized to determine the severity and prognosis of cirrhosis. Although the CTP score includes subjective variables such as ascites and hepatic encephalopathy, it maintains its prognostic value in the management of variceal bleeding [5]. On the other hand, the MELD score, comprising objective parameters such as serum creatinine, bilirubin, and INR, has become the standard for predicting mortality, particularly in organ transplant waiting lists. However, the traditional MELD score does not account for hyponatremia, which is an independent predictor of mortality in cirrhosis. To address this deficiency, the MELD-Na score, which integrates serum sodium levels into the formula, has been shown to be superior in predicting mortality, especially in patients with low MELD scores [5]. Conversely, it remains controversial whether these scores, which focus primarily on liver function, fully reflect hemodynamic instability and the systemic inflammatory response at the time of acute bleeding [6]. Consequently, clinical interest in non-invasive prognostic markers persists. PC/SD ratio, first proposed in the early 2000s, is a pathophysiologically based parameter that has been extensively studied for predicting the presence of varices [7,8].

PC/SD is a pathophysiologically based parameter that correlates hypersplenism with thrombocytopenia and has traditionally been used to predict the presence of high-risk esophageal varices [9,10]. Although the success of PC/SD ratio in variceal screening is well-established, studies investigating its role in predicting clinical outcomes such as rebleeding and mortality following acute hemorrhage are limited, and the results remain contradictory [11]. Similarly, it is known that infection and systemic inflammation have a triggering effect on treatment failure and mortality in AGEVB. NLR, which can be easily calculated from inexpensive and routine complete blood count (CBC) tests, is considered a robust indicator of the systemic inflammatory response [12]. High NLR values have been reported to be associated with poor prognosis and increased mortality in patients with decompensated cirrhosis, independent of the presence of infection [6]. However, the comparative value of NLR in predicting the prognosis of AGEVB, when used alongside liver-specific scores (MELD-Na, CTP) and portal hypertension markers (PC/SD), has not yet been fully elucidated.

In the current literature, studies providing a head-to-head comparison of these four distinct prognostic markers representing the functional reserve of the liver (CTP, MELD-Na), the indirect severity of portal hypertension (PC/SD), and the systemic inflammatory response (NLR) in patients with GEVB are limited. Although MELD-Na, Child–Pugh, NLR, and PC/SD have each been evaluated separately in previous studies, direct comparisons of these markers within the same AGEVB cohort remain scarce. Furthermore, most available studies have focused on either mortality or rebleeding alone, limiting assessment of their relative prognostic performance. Therefore, a comprehensive comparison of these commonly used prognostic markers may provide clinically relevant information for risk stratification and management of patients with AGEVB.

The aim of this study is to compare the performances of CTP, MELD-Na, PC/SD and NLR in predicting 6-week mortality and the risk of rebleeding in a cohort of patients diagnosed with acute esophageal variceal bleeding at a single center, and to identify the most useful prognostic marker for clinical practice.

## 2. Methods

### 2.1. Study Design and Setting

This retrospective, single-center cohort study was conducted at a tertiary referral hospital and included consecutive adult patients admitted with acute gastroesophageal variceal bleeding between January 2015 and June 2025. The study protocol was approved by the Ethics Committee of Ondokuz Mayıs University Faculty of Medicine (Approval Code: OMUKAEK No: 2026/20). The study was conducted in accordance with the Declaration of Helsinki. Due to the retrospective design, the requirement for written informed consent was waived by the ethics committee.

### 2.2. Study Population and Exclusion Criteria

During the study period, a total of 211 consecutive patients presenting with suspected AGEVB were retrospectively screened. Of these, 61 patients were excluded according to the following predefined criteria:

### 2.3. Patient Selection and Inclusion Criteria

1. Age: Adult patients aged 18 years and older.
2. Clinical Diagnosis: Patients with a confirmed diagnosis of liver cirrhosis based on clinical, laboratory, or radiological findings.
3. Index Bleeding Episode: The first (index) admission to the hospital during the study period due to acute gastroesophageal variceal bleeding (confirmed by nipple sign, active bleeding, or high-risk stigmata).
4. Endoscopic Confirmation: Patients diagnosed with AGEVB via upper gastrointestinal endoscopy, characterized by active spurting or oozing, an adherent clot on the varix, “cherry-red” spots, or a “nipple sign” (fibrin plug).
5. Standard Treatment: Patients were initiated on standard vasoactive therapy (terlipressin, somatostatin, or octreotide) immediately upon admission. Endoscopic intervention, including band ligation or cyanoacrylate injection, was performed within the first 12 h of presentation following hemodynamic stabilization.
6. Data Completeness: Patients with complete baseline laboratory and radiological data required for the calculation of the scoring systems (MELD-Na, Child-Pugh, NLR, PC/SD).

### 2.4. Exclusion Criteria

During the study period, 211 patient records evaluated for suspected acute gastroesophageal variceal bleeding were reviewed. Patients meeting any of the following criteria were excluded:

1. Duplicate Records: Subsequent admissions of the same patient within or after 6 weeks were not treated as independent cases; instead, they were evaluated as a “re-bleeding outcome” of the index admission. Therefore, repeat admissions were excluded as separate cases (*n* = 31).
2. Non-Cirrhotic Portal Hypertension: Patients with portal hypertension due to causes other than liver cirrhosis (e.g., portal vein thrombosis, idiopathic) (*n* = 7).
3. Cardiac Cirrhosis: Patients diagnosed with cardiac cirrhosis were excluded (*n* = 2). No other secondary causes of cirrhosis were identified in the excluded cohort.
4. Concomitant Malignancies: Patients diagnosed with hepatocellular carcinoma (*n* = 13), liver metastasis (*n* = 2), and acute myeloid leukemia (*n* = 1).
5. Missing Data: Patients whose clinical follow-up could not be completed or who had missing baseline laboratory and radiological data necessary for calculating prognostic scores (NLR, MELD-Na, Child-Pugh, PC/SD ratio) (*n* = 5).

**Figure 1:**
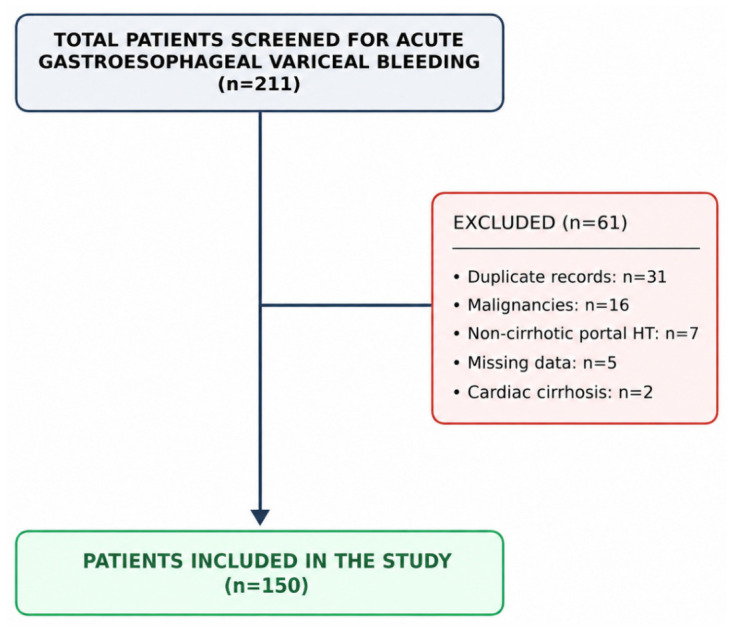
Study flow diagram showing the inclusion and exclusion criteria.

After application of these exclusion criteria, 150 patients constituted the final study cohort and were included in the statistical analyses (Figure 1).

### 2.5. Management of GEVB

Initial management focused on prompt hemodynamic resuscitation with crystalloids and blood products to maintain a target hemoglobin level of 7–9 g/dL. Intravenous vasoactive therapy (somatostatin or terlipressin) was started at admission and continued for 3 to 5 days. All patients received prophylactic broad-spectrum antibiotic prophylaxis according to institutional protocols and contemporary guideline recommendations. Third-generation cephalosporins, predominantly ceftriaxone, were the preferred first-line agents during the study period. Alternative antibiotic regimens were reserved for selected patients based on clinical indications, allergy status, or physician discretion. Emergency endoscopy was conducted within 12 h of admission. Esophageal varices were managed with endoscopic band ligation (EBL), while gastric varices were treated with cyanoacrylate (Histoacryl^®^) injection when feasible.

Post-bleeding management followed the recommendations of the Baveno VII consensus, including secondary prophylaxis with non-selective beta-blockers (NSBB) and/or repeated endoscopic band ligation. Information regarding the use of NSBB prior to the index bleeding episode was systematically collected. In patients not receiving NSBB at admission, therapy was initiated or titrated after hemodynamic stabilization.

By ensuring that all patients received guideline-based standard of care, the prognostic performance of Child–Pugh, MELD-Na, NLR, and PC/SD in this study reflects their ability to predict short-term outcomes despite optimal contemporary management.

### 2.6. Data Collection

Demographic characteristics (age, sex), etiology of cirrhosis, clinical presentation (hematemesis, melena, hematochezia), hemodynamic status at admission, laboratory parameters obtained at the time of index bleeding, and endoscopic findings were retrieved from electronic medical records. Laboratory variables used for score calculations were obtained from blood samples collected at initial presentation before endoscopic or pharmacological intervention.

The presence of infectious complications during hospitalization, including pneumonia and spontaneous bacterial peritonitis, was recorded based on clinical, laboratory, radiological, and microbiological criteria. Spleen diameter was measured by abdominal ultrasonography performed during the index admission. PC/SD was calculated by dividing the platelet count (×10^9^/L) by the maximum bipolar spleen diameter (mm).

### 2.7. Definitions

AGEVB was defined as hematemesis and/or melena with endoscopic confirmation of esophageal or gastric varices and evidence of active bleeding or stigmata of recent hemorrhage.

Varices were classified according to the Sarin classification as gastroesophageal varices (GOV1 and GOV2) or isolated gastric varices (IGV1 and IGV2) based on their anatomical location and extension.

Early rebleeding was defined as recurrent variceal hemorrhage occurring within 6 weeks after initial hemostasis and confirmed by endoscopy.

Six-week mortality was defined as death from any cause occurring within 42 days of the index variceal bleeding episode.

Hypotension was defined as systolic blood pressure < 90 mmHg at presentation or the requirement for vasopressor support.

Pneumonia was diagnosed based on new pulmonary infiltrates on chest imaging, together with clinical signs of infection and elevated inflammatory markers.

Spontaneous bacterial peritonitis was diagnosed by an ascitic fluid neutrophil count ≥ 250 cells/mm^3^ with or without positive culture, in the absence of an intra-abdominal surgically treatable source. Only pneumonia and spontaneous bacterial peritonitis were systematically recorded and analyzed as infectious complications in the present study. Other infectious conditions, including sepsis without a documented source, urinary tract infections, or bloodstream infections, were not consistently recorded in the retrospective database and were therefore not included in the analyses.

### 2.8. Prognostic Scores

Liver disease severity was determined using CTP and MELD-Na scores. Systemic inflammatory status was quantified via NLR, while PC/SD was employed as an indirect marker of portal hypertension severity.

### 2.9. Statistical Analysis

Continuous variables were expressed as mean ± standard deviation or median (interquartile range), as appropriate. Categorical variables were presented as numbers and percentages. Normality of distribution was assessed using the Kolmogorov–Smirnov and Shapiro–Wilk tests. Comparisons between two independent groups (rebleeding vs. no rebleeding; survivors vs. non-survivors) were performed using Student’s *t*-test or the Mann–Whitney U test for continuous variables and the χ^2^ test or Fisher’s exact test for categorical variables.

The diagnostic performance of CTP, MELD-Na, NLR and PC/SD in predicting 6-week mortality and early rebleeding was evaluated using receiver operating characteristic (ROC) curve analysis, and the area under the curve (AUC) with 95% confidence intervals was calculated. Pairwise comparisons of AUCs were performed using the DeLong method.

Optimal cut-off values for both Child–Pugh and MELD-Na scores were determined using the Youden index. Sensitivity, specificity, positive predictive value, and negative predictive value were calculated for each score.

Given the low number of mortality events, multivariable analysis was performed using penalized logistic regression to reduce small-sample bias and avoid model instability. The model included only prognostic scores (Child–Pugh, MELD-Na, NLR, and PC/SD) to identify independent predictors of 6-week mortality. Results were expressed as odds ratios (ORs) with 95% confidence intervals.

Survival analysis was performed using the Kaplan–Meier method, and differences between groups stratified according to the optimal cut-off value of the best prognostic score were compared using the log-rank test.

A two-sided *p* value < 0.05 was considered statistically significant. Statistical analyses were performed using IBM SPSS Statistics for Windows, version 26.0 (IBM Corp., Armonk, NY, USA) and R software, version R 4.5.3 (R Foundation for Statistical Computing, Vienna, Austria).

## 3. Results

### 3.1. Baseline Characteristics of the Study Population

A total of 150 patients with AGEVB were included in the final analysis. The baseline demographic, clinical, laboratory, and endoscopic characteristics of the study population are summarized in Table 1.

The mean age of the cohort was 60.7 ± 13.6 years. The most common etiologies of cirrhosis were hepatitis B virus infection (30.7%), cryptogenic cirrhosis (29.3%), metabolic dysfunction-associated steatotic liver disease (14.0%), and alcohol-related liver disease (13.3%). According to the Sarin classification, the majority of patients had GOV1 varices (93.3%), followed by GOV2 (4.7%) and IGV1 (2.0%).

At presentation, hematemesis was observed in 77.3% of patients, melena in 66.7%, and hematochezia in 7.3%. More than half of the cohort (56.7%) were receiving non-selective beta-blocker therapy at the time of the index bleeding episode.

The mean MELD-Na and CTP were 13.9 ± 6.6 and 7.2 ± 1.6, respectively. The mean NLR was 5.6 ± 5.0, and the mean PC/SD was 708.0 ± 548.3.

### 3.2. Comparison According to Early Rebleeding

Early rebleeding within 6 weeks after the index variceal hemorrhage occurred in 30 patients (20.0%), whereas 120 patients (80.0%) did not experience rebleeding. Comparisons of baseline characteristics according to rebleeding status are presented in Table 2.

Patients who developed early rebleeding had significantly higher liver disease severity scores at admission. Both the MELD-Na score (15.7 ± 7.1 vs. 13.4 ± 6.4, *p* = 0.039) and CTP (7.9 ± 1.9 vs. 7.0 ± 1.5, *p* = 0.011) were significantly higher in the rebleeding group. In contrast, systemic inflammatory status assessed by NLR (5.8 ± 4.2 vs. 5.6 ± 5.2, *p* = 0.674) and portal hypertension severity assessed by PC/SD (683.8 ± 425.8 vs. 713.8 ± 575.2, *p* = 0.718) did not differ significantly between groups.

Regarding clinical presentation, melena was more frequently observed in patients who experienced rebleeding (86.7% vs. 61.7%, *p* = 0.019), whereas the rates of hematemesis and hematochezia were similar between groups. Variceal type according to the Sarin classification, use of non-selective beta-blockers, and underlying etiology of cirrhosis were not significantly associated with the occurrence of early rebleeding.

**Figure 2:**
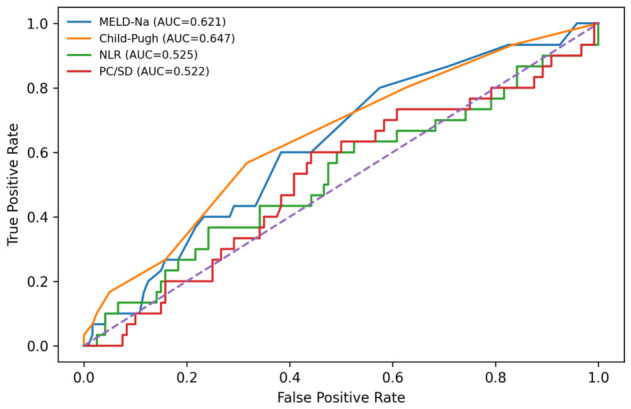
ROC curves of Child–Pugh score, MELD-Na score, NLR, and PC/SD for prediction of early rebleeding within 6 weeks after the index bleeding episode. ROC: Receiver Operating Characteristic.

**Figure 3:**
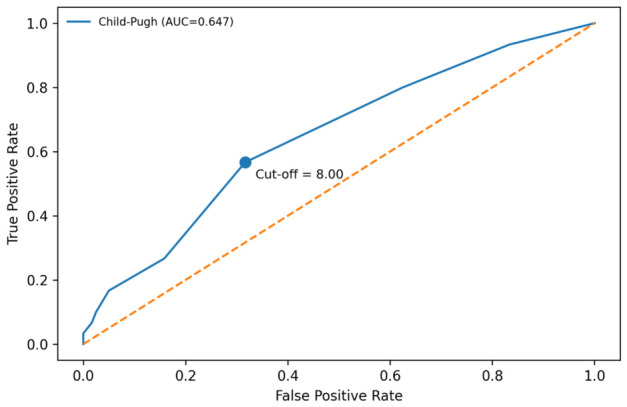
Determination of the optimal cut-off value of the Child–Pugh score for prediction of early rebleeding using the Youden index.

The discriminative performance of CTP, MELD-Na, NLR, and PC/SD for predicting early rebleeding is illustrated by ROC curve analysis in Figure 2. Among these parameters, CTP demonstrated the highest area under the curve. Accordingly, the optimal CTP cut-off value for predicting early rebleeding was determined using the Youden index (Figure 3).

### 3.3. Six-Week Mortality and Comparison Between Survivors and Non-Survivors

Overall, 17 patients (11.3%) died within 6 weeks of the index variceal bleeding episode. Comparisons between survivors and non-survivors are summarized in Table 3.

Patients who died within 6 weeks had significantly more advanced liver disease at presentation. Both MELD-Na score (21.0 ± 8.9 vs. 13.0 ± 5.7, *p* = 0.0006) and Child–Pugh score (9.2 ± 2.2 vs. 6.9 ± 1.3, *p* < 0.0001) were markedly higher in non-survivors. In contrast, NLR (7.7 ± 10.7 vs. 5.3 ± 3.6, *p* = 0.626) and PC/SD (751.1 ± 597.8 vs. 691.5 ± 524.7, *p* = 0.900) did not differ significantly between groups.

Clinical complications were substantially more frequent among non-survivors. The prevalence of pneumonia (29.4% vs. 8.4%, *p* = 0.027), spontaneous bacterial peritonitis (17.6% vs. 0.8%, *p* = 0.001), and hypotension at presentation (76.5% vs. 21.4%, *p* < 0.0001) was significantly higher in patients who died within 6 weeks. Variceal type, use of non-selective beta-blockers, and presenting symptoms were not significantly associated with mortality.

### 3.4. ROC Analysis for Prediction of 6-Week Mortality

**Figure 4:**
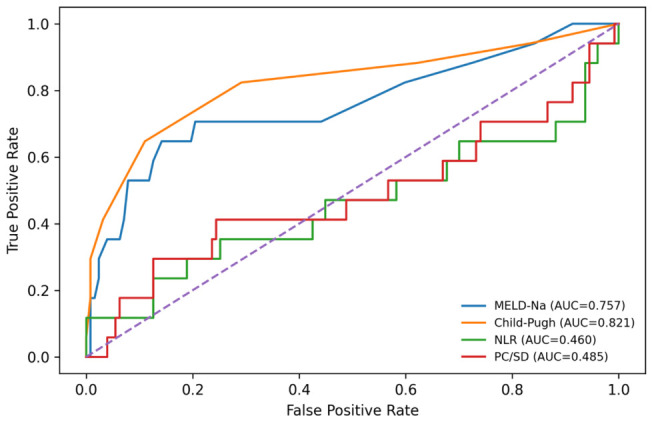
Receiver operating characteristic (ROC) curves of Child–Pugh score, MELD-Na score, neutrophil-to-lymphocyte ratio (NLR), and platelet count-to-spleen diameter ratio (PC/SD) for prediction of 6-week mortality.

ROC curve analysis was employed to evaluate the predictive performance of CTP, MELD-Na, NLR, and PC/SD regarding 6-week mortality (Figure 4). CTP demonstrated the highest area under the ROC curve (AUROC), surpassing MELD-Na. In contrast, both NLR and PC/SD showed modest discriminative ability.

**Figure 5:**
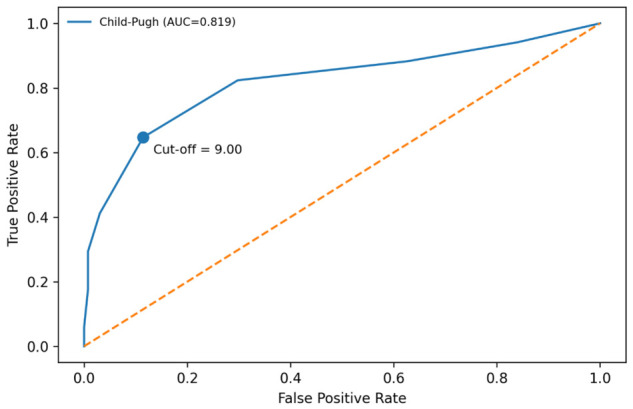
Determination of the optimal cut-off value of the Child–Pugh score for prediction of 6-week mortality using the Youden index.

Pairwise comparison of AUCs revealed that the AUC of CTP was significantly higher than those of NLR and PC/SD, whereas the difference between CTP and MELD-Na was not statistically significant. Using the Youden index, the optimal cut-off values for predicting 6-week mortality were identified as 9 for Child–Pugh and 19 for MELD-Na. The MELD-Na threshold of 19 yielded a sensitivity of 64.7% and a specificity of 85.5%. Given its superior discriminative performance, the Child–Pugh cut-off was subsequently used for survival analyses (Figure 5).

### 3.5. Survival Analysis

**Figure 6:**
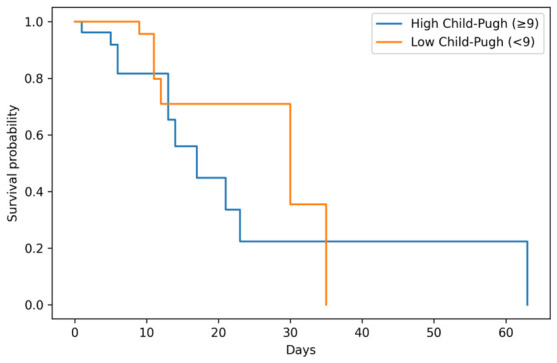
Kaplan–Meier survival curves according to the optimal Child–Pugh cut-off value for 6-week mortality. Survival distributions were compared using the log-rank test.

Kaplan–Meier survival curves stratified according to the optimal CTP cut-off value are shown in Figure 6. Patients with higher CTP exhibited significantly lower 6-week survival compared with those with lower scores. The difference in survival between the two groups was statistically significant by the log-rank test, indicating a strong association between advanced liver dysfunction and short-term mortality after acute variceal bleeding.

### 3.6. Multivariate Analysis

**Figure 7:**
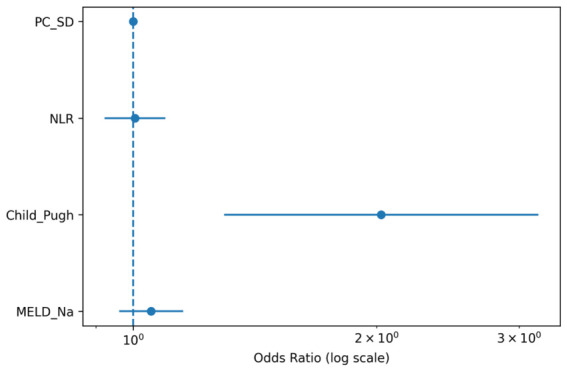
Forest plot of penalized multivariate logistic regression including prognostic scores (Child–Pugh, MELD-Na, NLR, and PC/SD) for prediction of 6-week mortality. Odds ratios (ORs) with 95% confidence intervals are shown.

Given the limited number of mortality events, a penalized multivariate logistic regression model including only prognostic scores (CTP, MELD-Na, NLR, and PC/SD) was constructed to reduce small-sample bias and avoid model instability. As shown in Figure 7, CTP remained an independent predictor of 6-week mortality, whereas NLR and PC/SD did not demonstrate independent prognostic significance after adjustment. MELD-Na showed a weaker association and did not retain independent significance when analyzed together with CTP. Multivariable estimates should be interpreted cautiously due to the limited number of events.

## 4. Discussion

This retrospective cohort study compared the prognostic performance of scores reflecting the severity of hepatic dysfunction (CTP and MELD-Na) and non-invasive markers of systemic inflammation and portal hypertension (NLR and PC/SD) in predicting clinical outcomes in cirrhotic patients presenting with AGEVB. The main finding of our study is that CTP and MELD-Na scores demonstrated high discriminative ability for 6-week mortality, whereas NLR and PC/SD failed to show sufficient prognostic performance as independent predictors of either mortality or early rebleeding in multivariable analysis.

Patients who died within 6 weeks had significantly higher CTP and MELD-Na scores, supporting the concept that prognosis in acute variceal hemorrhage is primarily determined by the underlying hepatic functional reserve rather than by the acute severity of bleeding itself. This observation is in line with previous reports. Pham et al. showed, in a prospective cohort, that MELD and its derivatives outperformed bleeding-specific scores such as AIMS65 and the Glasgow–Blatchford score in predicting 6-week mortality [13]. Similarly, Aluizio et al. reported that MELD and CTP were accurate predictors of mortality but had limited value for forecasting rebleeding [14]. Our findings further emphasize the added prognostic value of serum sodium incorporated into MELD-Na, particularly in decompensated patients at risk for ascites and hepatorenal syndrome [15]. Consistently, Nguyen et al. recently demonstrated that CTP class C and elevated serum creatinine were independent risk factors for in-hospital mortality [16], reinforcing the pivotal role of hepatic and renal dysfunction in outcome prediction.

Systemic inflammation is known to be associated with adverse outcomes in cirrhosis. Chen et al. (2024) identified elevated NLR as an independent predictor of in-hospital mortality in upper gastrointestinal bleeding [12], while Cazacu et al. reported that NLR predicted very early (48 h) mortality but lost accuracy for mid-term (6-week) outcomes [6]. In our cohort, although NLR values were numerically higher among non-survivors, NLR did not emerge as an independent prognostic factor in multivariable analysis. An important methodological aspect of our study is that NLR was calculated at baseline, prior to the initiation of vasoactive agents or antibiotics that might influence leukocyte kinetics. Lagadinou et al. showed that baseline NLR correlates with MELD score but that its prognostic value is strongly modulated by the presence of infection [17]. Because infection was not an exclusion criterion in our study, inflammation related to concomitant infections may have masked the bleeding-specific prognostic signal of NLR. Our results suggest that the systemic inflammatory response triggered by acute bleeding, as reflected by NLR, loses statistical strength when evaluated alongside robust composite scores integrating hepatic and renal dysfunction, such as MELD-Na.

Another noteworthy finding is the limited prognostic value of the PC/SD ratio, a parameter widely used for non-invasive prediction of varices, in forecasting “hard” outcomes such as mortality and rebleeding after an acute hemorrhagic event. Several studies, including those by González-Ojeda et al. [7] and Jamil et al. [8], have demonstrated high accuracy of PC/SD for variceal screening. However, its utility in the acute bleeding setting remains controversial. Although Liu et al. (2022) reported the PC/spleen thickness ratio as an independent predictor of rebleeding [11], we observed no significant difference in PC/SD between survivors and non-survivors. This discrepancy may be explained by the effects of acute hemodynamic instability and aggressive fluid resuscitation on platelet concentration, thereby reducing the predictive reliability of this ratio. Moreover, Mattos et al. noted that although PC/SD performs excellently for variceal detection, it is not consistently robust as an independent prognostic variable [18]. Our findings indicate that PC/SD is a valuable screening tool in stable cirrhotic patients but may be insufficient for risk stratification during acute variceal bleeding.

Early rebleeding is a major determinant of mortality in cirrhotic patients. In our cohort, rebleeding episodes were managed according to current guidelines, primarily using repeat endoscopic band ligation or hemostatic agents. Balloon tamponade was not utilized as a standard treatment, reflecting the shift toward more advanced endoscopic and radiological interventions in contemporary practice. In our study, rebleeding rates were comparable to those reported in the literature and were closely associated with impaired hepatic reserve. Salman et al. found a 28% rebleeding rate during 3-year follow-up and identified CTP class C as an independent predictor of rebleeding [19]. Similarly, Matei et al. demonstrated that CTP class C status was independently associated with failure to control bleeding and increased mortality [20]. The modest performance of prognostic scores for rebleeding in our cohort suggests that rebleeding is influenced not only by biochemical and clinical parameters but also by factors not captured in our analysis, such as endoscopic therapy quality and portal pressure gradients (HVPG).

Regarding the etiology of cirrhosis, the high prevalence of cryptogenic cases (29.3%) in our study is noteworthy. This can be attributed to several factors: first, a significant portion of these cases likely represents ‘burnt-out’ metabolic dysfunction-associated steatotic liver disease (MASLD), where typical histological or radiological features were no longer present at the time of diagnosis. Second, in our sociocultural context, patients may underreport alcohol consumption, leading to an overestimation of cryptogenic cases. Conversely, the low prevalence of HCV-related cirrhosis (2.7%) reflects the successful implementation of highly effective direct-acting antiviral (DAA) therapies in our national healthcare system over the last decade, which has significantly reduced the burden of HCV-induced end-stage liver disease.

Several limitations should be acknowledged. First, the retrospective, single-center design may limit generalizability. Second, the absence of routine HVPG measurements precluded assessment of the relationship between hemodynamic severity of portal hypertension and non-invasive markers. Third, inclusion of patients with concomitant infections may have reduced the specificity of NLR; however, this approach reflects a real-world emergency department population and enhances external validity. From a practical perspective, Child–Pugh and MELD-Na may serve complementary roles in clinical decision-making. The Child–Pugh score can be rapidly calculated at the bedside and demonstrated the highest discriminative performance in our cohort, making it suitable for early risk stratification. In contrast, MELD-Na provides a more objective assessment based exclusively on laboratory variables and may be particularly useful for identifying patients who require closer monitoring, intensive care support, or consideration for advanced therapies. Together, these scores may complement existing management algorithms and facilitate individualized patient care.

## 5. Conclusions

In conclusion, our study demonstrates that MELD-Na and CTP are reliable prognostic indicators not only for 6-week mortality but also for early rebleeding risk in patients with AGEVB. While these scores reflecting hepatic functional reserve were significantly higher in patients who experienced rebleeding, NLR and PC/SD showed no discriminative value for either rebleeding or mortality. These findings suggest that prognostic assessment in acute variceal bleeding should primarily focus on the severity of liver failure rather than on indirect markers of inflammation or splenic size. In clinical practice, patients with high MELD-Na and CTP should be regarded as a high-risk group for both early rebleeding and death and should therefore undergo closer monitoring and more aggressive management strategies.

## Figures and Tables

**Table 1:** Baseline Characteristics of the Study Population (*n* = 150).

Variable	Total Cohort (*n* = 150)
Age, mean ± SD (years)	60.7 ± 13.6
MELD-Na score, mean ± SD	13.9 ± 6.6
Child-Pugh score, mean ± SD	7.2 ± 1.6
NLR, mean ± SD	5.6 ± 5.0
PC/SD, mean ± SD	708.0 ± 548.3
Sarin classification, *n* (%)	
GOV1	140 (93.3)
GOV2	7 (4.7)
IGV1	3 (2.0)
NSBB use, *n* (%)	85 (56.7)
Clinical presentation, *n* (%)	
Hematemesis	116 (77.3)
Melena	100 (66.7)
Hematochezia	11 (7.3)
Etiology of cirrhosis, *n* (%)	
HBV	46 (30.7)
Cryptogenic	44 (29.3)
Alcohol-related	20 (13.3)
MASLD	21 (14.0)
Autoimmune hepatitis	7 (4.7)
HCV	4 (2.7)
Other causes	8 (5.3)

Baseline demographic, clinical, laboratory, and endoscopic characteristics of the study population (*n* = 150). Values are presented as mean ± standard deviation or number (percentage), as appropriate. NLR, neutrophil-to-lymphocyte ratio; PC/SD, platelet count-to-spleen diameter ratio; NSBB, non-selective beta-blocker; GOV, gastroesophageal varices; IGV, isolated gastric varices.

**Table 2:** Comparison According to Early Rebleeding (Within 6 Weeks).

Variable	No Rebleeding (*n* = 120)	Rebleeding (*n* = 30)	*p*-Value
Age, mean ± SD (years)	60.5 ± 13.9	61.3 ± 12.6	0.5888
MELD-Na, mean ± SD	13.4 ± 6.4	15.7 ± 7.1	0.039 *
Child-Pugh, mean ± SD	7.0 ± 1.5	7.9 ± 1.9	0.011 *
NLR, mean ± SD	5.6 ± 5.2	5.8 ± 4.2	0.6741
PC/SD, mean ± SD	713.8 ± 575.2	683.8 ± 425.8	0.7175
Sarin classification			0.3451
GOV1, *n* (%)	111 (92.5)	29 (96.7)	
GOV2, *n* (%)	7 (5.8)	0 (0.0)	
IGV1, *n* (%)	2 (1.7)	1 (3.3)	
NSBB use (Propranolol or Carvedilol), *n* (%)	63 (52.5)	22 (73.3)	0.0703
Hematemesis, *n* (%)	93 (77.5)	23 (76.7)	1.000
Melena, *n* (%)	74 (61.7)	26 (86.7)	0.019 *
Hematochezia, *n* (%)	11 (9.2)	0 (0.0)	0.1803
Etiology			0.6273
HBV, *n* (%)	39 (32.5)	7 (23.3)	
Cryptogenic, *n* (%)	33 (27.5)	11 (36.7)	
Alcohol-related, *n* (%)	14 (11.7)	6 (20.0)	
MASLD, *n* (%)	19 (15.8)	2 (6.7)	
Autoimmune hepatitis, *n* (%)	6 (5.0)	1 (3.3)	
HCV, *n* (%)	3 (2.5)	1 (3.3)	
Other causes, *n* (%)	6 (5.0)	2 (6.7)	

Comparison of baseline characteristics according to early rebleeding within 6 weeks after the index variceal hemorrhage. Continuous variables are expressed as mean ± standard deviation and categorical variables as numbers (percentages). Statistically significant *p* values (<0.05) are indicated in bold. NLR, neutrophil-to-lymphocyte ratio; PC/SD, platelet count-to-spleen diameter ratio; NSBB, non-selective beta-blocker. * statistically significant.

**Table 3:** Comparison of survivors and non-survivors with acute variceal bleeding.

Variable	Survivors (*n* = 131)	Non-Survivors (*n* = 17)	*p*-Value
**Age (mean ± SD)**	60.0 ± 13.3	65.4 ± 15.9	0.1302
**MELD-Na (mean ± SD)**	13.0 ± 5.7	21.0 ± 8.9	0.0006 *
**Child-Pugh (mean ± SD)**	6.9 ± 1.3	9.2 ± 2.2	<0.0001 *
**NLR (mean ± SD)**	5.3 ± 3.6	7.7 ± 10.7	0.6262
**PC/Spleen Diameter (mean ± SD)**	691.5 ± 524.7	751.1 ± 597.8	0.8995
**Pneumonia, *n* (%)**	11 (8.4)	5 (29.4)	0.0271 *
**Spontaneous bacterial peritonitis, *n* (%)**	1 (0.8)	3 (17.6)	0.0012 *
**Hypotension, *n* (%)**	28 (21.4)	13 (76.5)	<0.0001 *
**Sarin classification**			0.8006
GOV1, *n* (%)	122 (93.1)	16 (94.1)	
GOV2, *n* (%)	6 (4.6)	1 (5.9)	
IGV1, *n* (%)	3 (2.3)	0 (0.0)	
**NSBB use, *n* (%)**	74 (56.5)	10 (58.8)	1.0000
**Presentation**			
Hematemesis, *n* (%)	103 (78.6)	11 (64.7)	0.2980
Melena, *n* (%)	84 (64.1)	15 (88.2)	0.0934
Hematochezia, *n* (%)	9 (6.9)	2 (11.8)	0.8232
**Etiology**			0.1277
HBV, *n* (%)	41 (31.3)	4 (23.5)	
Cryptogenic, *n* (%)	40 (30.5)	4 (23.5)	
Alcohol, *n* (%)	14 (10.7)	6 (35.3)	
MASLD, *n* (%)	19 (14.5)	1 (5.9)	
Autoimmune hepatitis, *n* (%)	6 (4.6)	1 (5.9)	
HCV, *n* (%)	3 (2.3)	1 (5.9)	
Other, *n* (%)	8 (6.1)	0 (0.0)	

Comparison of clinical, laboratory, and prognostic parameters between survivors and non-survivors within 6 weeks. Continuous variables are expressed as mean ± standard deviation and categorical variables as numbers (percentages). NLR, neutrophil-to-lymphocyte ratio; PC/SD, platelet count-to-spleen diameter ratio; SBP, spontaneous bacterial peritonitis. * Statistically significant.

## Data Availability

The data presented in this study are available from the corresponding author upon reasonable request. The data are not publicly available due to institutional and ethical restrictions.

## Abbreviations

AGEVB, acute gastroesophageal variceal bleeding; CTP, Child–Pugh score; MELD-Na, Model for End-Stage Liver Disease–Sodium; NLR, neutrophil-to-lymphocyte ratio; PC/SD, platelet count-to-spleen diameter ratio; ROC, receiver operating characteristic; AUC, area under the curve; NSBB, non-selective beta-blocker; SBP, spontaneous bacterial peritonitis; GOV, gastroesophageal varices; IGV, isolated gastric varices; HBV, hepatitis B virus; HCV, hepatitis C virus; MASLD, metabolic dysfunction-associated steatotic liver disease; HVPG, hepatic venous pressure gradient.
